# Study on the prevalence and genetic diversity of *Eimeria* species from broilers and free-range chickens in KwaZulu-Natal province, South Africa

**DOI:** 10.4102/ojvr.v87i1.1837

**Published:** 2020-09-17

**Authors:** Abiodun J. Fatoba, Oliver T. Zishiri, Damer P. Blake, Sunday O. Peters, Jeffrey Lebepe, Samson Mukaratirwa, Matthew A. Adeleke

**Affiliations:** 1 Discipline of Genetics, School of Life Sciences, College of Agriculture, Engineering and Sciences, University of KwaZulu-Natal, Westville, South Africa; 2 Department of Pathobiology and Population Sciences, The Royal Veterinary College, Hawkshead Lane, Hertfordshire, United Kingdom; 3 Department of Animal Science, Berry College, Mount Berry, Georgia, United States; 4 Department of Biodiversity and Evolutionary Biology, School of Life Sciences, College of Agriculture, Engineering and Sciences, University of KwaZulu-Natal, Durban, South Africa; 5 Department of Biological Sciences, School of Life Sciences, College of Agriculture, Engineering and Sciences, University of KwaZulu-Natal, Durban, South Africa

**Keywords:** chickens, coccidiosis, *Eimeria*, genetic diversity, molecular diagnosis, prevalence

## Abstract

This study was conducted from January to October 2018 with the objective to determine the prevalence and genetic diversity of *Eimeria* species in broiler and free-range chickens in KwaZulu-Natal province, South Africa. A total of 342 faecal samples were collected from 12 randomly selected healthy broiler chicken farms and 40 free-range chickens from 10 different locations. Faecal samples were screened for the presence of *Eimeria* oocysts using a standard flotation method. The species of *Eimeria* isolates were confirmed by amplification of the internal transcribed spacer 1 (ITS-1) partial region and sequences analysis. Among broiler and free-ranging chickens, 19 out of 41 pens (46.3%) and 25 out of 42 faecal samples (59.5%) were positive for *Eimeria* infection. Molecular detection revealed the following species: *Eimeria maxima, Eimeria tenella, Eimeria acervulina, Eimeria brunetti* and *Eimeria mitis* in all the samples screened. Similarly, polymerase chain reaction assays specific for three cryptic *Eimeria* operational taxonomic units were negative for all the samples. Phylogenetic analysis of the ITS-1 sequences supported species identity with the greatest variation detected for *E. mitis*. This study provides information on the range and identity of *Eimeria* species, and their genetic relatedness, circulating in commercially reared broilers and free-ranging chickens from different locations in KwaZulu-Natal province.

## Introduction

Poultry production has become a major driving force in the economy of many developing countries, which are countries characterised by low income and gross domestic product per capita (Alders & Pym 2009). South Africa produced 129.3 million chickens throughout the nine provinces in 2017, of which 7% were from KwaZulu-Natal (South African Poultry Association 2017). The vulnerability of chickens under commercial production to parasitic diseases such as coccidiosis is a major threat to the productivity and viability of the South African poultry industry.

Coccidiosis is an enteric disease that reduces performance and affects the welfare of chickens, leading to high morbidity and mortality in the absence of effective control (Blake & Tomley 2014). Globally, the annual burden of preventing/controlling coccidiosis has been estimated to exceed $3 billion (Blake & Tomley 2014). *Eimeria,* a parasite of the phylum Apicomplexa, is the causative agent of this disease, and its species such as *Eimeria necatrix, Eimeria maxima, Eimeria acervulina, Eimeria praecox, Eimeria mitis, Eimeria brunetti* and *Eimeria tenella* are also known to infect chickens (Nematollahi, Moghaddam & Niyazpour 2008). Mixed infections are common (Haug et al. 2008; Jenkins et al. 2008), thereby complicating diagnosis and effective control. The emergence of three cryptic *Eimeria* genotypes, referred to as operational taxonomic units (OTUs) x, y and z, has added further complexity. These were first detected circulating among commercial chickens reared in Australia (Cantacessi et al. 2008). The three OTU genotypes have since been reported in several African countries, such as Nigeria, Tanzania, Ghana, Uganda and Zambia (Clark et al. 2016; Jatau et al. 2016). The widespread occurrence of these cryptic genotypes could pose a significant risk to vaccine development and application (Clark et al. 2016).

Effective control of coccidiosis in chickens relies on strict management practices, supplemented by timely application of anticoccidial drugs and/or vaccines (Godwin & Morgan 2015) underpinned by proper diagnosis and identification. Traditional diagnostic methods include evaluation of the location and the characteristics of gross pathology (lesion scoring) and microscopic analysis of oocyst morphology **(**Kumar et al. 2014). However, the relative complexity and requirement of expertise for these methods necessitated the development of molecular alternatives, including genus- and species-specific polymerase chain reaction (PCR) assays (Lew et al. 2003). The use of nuclear and mitochondrial genetic markers (e.g. internal transcribed spacer [ITS] sequences, 18S ribosomal RNA, cytochrome oxidase subunit I [COI]) has proven effective in the identification and taxonomic classification of protozoan parasites, including *Eimeria* (Kumar et al. 2015a; Ogedengbe et al. 2018; Tan et al. 2017).

Thus, ITS-1 sequences have served as genetic markers to identify *Eimeria* species (Cook et al. 2010; Oliveira et al. 2011). Based on the observed diversity, ITS-based species-specific primers have been developed for use in the identification of *Eimeria* species (Lew et al. 2003). However, studies from various countries have reported nucleotide variations in the ITS-1 region within *Eimeria* species isolates (Bhaskaran et al. 2010; Kumar et al. 2015a; Lew et al. 2003). Genetic diversity among species and strains of *Eimeria* could pose a major risk to the control of coccidiosis in the future. As such, knowledge defining naturally occurring genetic diversity becomes imperative to understand the pathogenicity and epidemiology of *Eimeria* that infect chickens (Morris & Gasser 2006).

There is a dearth of information on *Eimeria* occurrence and diversity in South Africa. As such, reports on circulating *Eimeria* species in KwaZulu-Natal province together with information on their occurrence in commercial chickens are not available. This study, therefore, aimed to determine prevalence and genetic diversity of *Eimeria* species in both broiler and free-range chickens in KwaZulu-Natal province.

## Materials and methods

### Study area

KwaZulu-Natal is the second most populous province among the nine provinces in South Africa. It has a population of approximately 10 million people and land size of 94 000 km^2^ located between latitude 28°99’S and longitude 30°97’E. The capital city of Pietermaritzburg has a warm and subtropical climate throughout the year, especially around the coastline, but gets colder in the inland areas. The poultry industry in KwaZulu-Natal province is one of the producers of broiler birds in South Africa with a total of 6.7 million broiler birds in 2017, contributing 6.4% to the national broiler production (South African Poultry Association 2017).

### Sample collection

A total of 342 chicken faecal samples were collected from 12 broiler farms consisting of 41 pens (1–5 pens per farm) and free-range chickens. The age of broiler chickens at the time of sampling ranged from 3 to 10 weeks, with the exception of a single farm consisting of 12-week-old chickens. In addition, 42 faecal samples of 40 free-ranging 3-week-old village chickens were randomly collected from four localities. The 342 samples were collected randomly once from the following locations: Pietermaritzburg, Phoenix, Scottburg, Stanger, Chatsworth, Westville, Maphumulo, Umvoti, Port Sherpstone and Shongweni of KwaZulu-Natal province from January to October 2018. Detailed information on the number of pens per farm, number of samples per pen, number of farms per location and number of chickens per location is shown in Appendix 1 Tables 1-A1 and 2-A1. There were no clinical signs of coccidiosis among the chickens on any of the farms sampled. Samples were collected following the procedure described by Kumar et al. (2014). Briefly, in the broiler farms, faecal samples were collected following a pre-determined ‘W’ pathway in each pen to allow random sampling. Fifty-millilitre conical tubes containing 10 mL of 2% potassium dichromate were used to collect faeces up to 20 mL of the tube and stored at 4 °C until further use. Depending on the size of the pen, four to eight 50-mL conical tubes of faecal samples were collected per pen and the content was mixed together vigorously.

### Sample processing and microscopic oocyst identification

Samples were processed based on the procedures described by Kumar et al. (2014), with minor modifications. Two grams of faecal samples were weighed into a beaker and mixed with 100 mL of distilled water. This was stirred with a glass rod and later filtered through a gauze. The filtrate was transferred into a new 50-mL conical tube and filled to the brim with saturated salt solution. This was then centrifuged at 800 × g for 10 minutes. The supernatant was decanted and the sediment was transferred into a new 50-mL tube and then later pelleted at 14 000 × g for 3 min. Oocysts per gram (OPG) were counted using a McMaster counting chamber following a standard protocol (Haug, Williams & Larsen 2006). Samples with OPG greater or equal to 250 OPG were selected for deoxyribonucleic acid (DNA) extraction. Photomicrograph images of unsporulated oocysts were taken randomly from each farm sampled using an OMAX compound microscope containing a 5 MP camera at 400×.

### DNA extraction

Total genomic DNA was extracted using a Quick-DNA^TM^ Fecal/Soil Microbe Miniprep Kit (Zymo Research, United States [US]) based on the manufacturer’s protocol with minor modifications. Faecal samples in the Bashing Beads^TM^ lysis tube (0.1 mm and 0.5 mm) were processed on a Vortex Genie at maximum speed for 25 min, instead of 20 min as recommended by the manufacturer’s protocol. DNA quality and concentration were checked on an agarose gel (1.5%) and Nanodrop^TM^ 1000 spectrophotometer (Thermo Scientific, US) at 260 nm absorbance.

### Polymerase chain reaction amplification

A nested PCR protocol targeting the genomic ITS-1 region was used to detect each *Eimeria* species. Genus- and species-specific primers were used as described by Lew et al. (2003). Each 25 *µ*L PCR contained 12.5 *µ*L 2X DreamTaq Green PCR Master Mix (Thermo Scientific, US), 1 *µ*L of each forward and reverse primer (10 *µ*M of stock solution; Table 1), 5.5 *µ*L nuclease free water and 5 *µ*L DNA template. Thermal cycling was done as follows: initial denaturation at 94 °C for 3 min, 30 cycles of 94 °C for 30 s, 56 °C for 30 seconds and 72 °C for 90 s and a final extension at 72 °C for 15 min. The primary PCR product (1 *µ*L of the 25 *µ*L) was used as template for the nested PCR containing species-specific primers in each tube. The same thermal cycling conditions were used for the species with varying annealing temperature as follows: 55 °C for *E. mitis*, 56.7 °C for *E. tenella*, 61 °C for *E. acervulina*, 62 °C for *E. maxima*, 61 °C for *E. necatrix*, 61 °C for *E. praecox* and 61 °C for *E. brunetti*. Nuclease-free water replaced the DNA template for the negative control. Amplification of nested PCR products was checked on 1.5% (w/v) agarose gel at 100 V for 30 min and visualised under ultraviolet light using a Bio-Rad ChemiDoc^TM^ MP System (Bio-Rad, US). Similarly, the samples were also screened for the presence of three cryptic *Eimeria* OTUs by targeting the ITS-2 genomic region using the primers and thermal cycling procedure described by Fornace et al. (2013), as shown in Table 2. The PCR products were sent for sequencing at Inqaba Biotech (South Africa). Sequencing was done with both forward and reverse primers using Big Dye chemistries in an ABI 3500XL Genetic Analyzer, POP-7^TM^ (Thermo Scientific, US).

**TABLE 1:** Genus- and species-specific internal transcribed spacer-1 primers used in the study.

Genus-species	Primer strand	Primers	Annealing temperature (°C)	Length (bp)
*Eimeria* genus	Forward	AAGTTGCGTAAATAG AGCCCTC	56.0	Variable
	Reverse	AGACATCCATTGCTG AAAG		
*Eimeria tenella*	Forward	AATTTAGTCCATCGC AACCCT	56.7	278
	Reverse	CGAGCGCTCTGCATA CGACA		
*Eimeria acervulina*	Forward	GGC TTGGATGATGTT TGCTG	61.0	321
	Reverse	CGAACGCAATAACAC ACGCT		
*Eimeria brunetti*	Forward	GATCAG TTTGAGCAA ACCTTCG	61.0	311
	Reverse	TGGTCT TCCGTACGT CGGAT		
*Eimeria maxima*	Forward	CTACACCACTCAC AATGAGGCAC	62.0	145
	Reverse	GTGATATCGTTCTG GAGAAGTT TGC		
*Eimeria mitis*	Forward	GGGTTTATTTCCTGT CCGTCGTCTC	55.0	328
	Reverse	GCAAGAGAGAATCGG AATGCC		
*Eimeria praecox*	Forward	CCAAGCGATTTCATC ATTCGGGGAG	61.0	116
	Reverse	AAAAGCAACAGCGA TTCAAG		
*Eimeria necatrix*	Forward	TACATCCCAATCTTT GAATCG	61.0	383
	Reverse	GGCATACTAGCTTCG AGCAAC		

*Source:* Lew, A.E., Anderson, G.R., Minchin, C.M., Jeston, P.J. & Jorgensen, W.K., 2003, ‘Inter-and intra-strain variation and PCR detection of the internal transcribed spacer 1 (ITS-1) sequences of Australian isolates of *Eimeria* species from chickens’, *Veterinary Parasitology* 112(1–2), 33–50. https://doi.org/10.1016/S0304-4017(02)00393-X

Primers were all designed by Lew et al. (2003).

**TABLE 2:** Primers used for the detection of three cryptic *Eimeria* operational taxonomic units.

Species	Primer ref	Primer sequences	Annealing temperature (°C)	Size (bp)
OTUx	OTU_X_f1	GTGGTGTCGTCTGCGCGT	56	133
	OTU_X_r1	ACCACCGTATCTCTTTCGTGA		
OTUy	OTU_Y_f1	CAAGAAGTACACTACCACAGCATG	56	346
	OTU_Y_r1	ACTGATTTCAGGTCTAAAACGAAT		
OTUz	OTU_Z_f1	TATAGTTTCTTTTGCGCGTTGC	56	147
	OTU_Z_r1	CATATCTCTTTCATGAACGAAAGG		

*Source:* Lew, A.E., Anderson, G.R., Minchin, C.M., Jeston, P.J. & Jorgensen, W.K., 2003, ‘Inter-and intra-strain variation and PCR detection of the internal transcribed spacer 1 (ITS-1) sequences of Australian isolates of *Eimeria* species from chickens’, *Veterinary Parasitology* 112(1–2), 33–50. https://doi.org/10.1016/S0304-4017(02)00393-X

Primers were all designed by Fornace et al. (2013).

OTUs, operational taxonomic units; bp, base pair.

### Sequence analysis

A total of 28 ITS-1 sequences were viewed, edited and trimmed. Consensus sequences were generated from both forward and reverse sequences using BioEdit version 7.0.5.3 software (Hall 1999). The sequences were submitted to National Center Biotechnology Information and assigned accession numbers (Appendix 1 Table 3-A1). Also, the sequences were compared with selected published sequences from the GenBank. Sequence alignment was performed using the ClustalW programme. Pairwise percentage identity (Appendix 1 Figure 1-A1) was carried using Sequence Demarcation Tool (SDT) version 1.2 software (Muhire, Varsani & Martin 2014). Genetic distance within *Eimeria* species isolates from this study was calculated with MEGA version 6.0 (Tamura et al. 2013) using the Tamura 3-parameter model.

### Phylogenetic analysis of internal transcribed spacer-1 sequences

The genetic diversity that exists between the ITS-1 sequences generated in this study (*n* = 28) and those of American, Chinese, Indian, Australian, Egypt, Sudan and Swedish *Eimeria* species isolates published in GenBank (Appendix 1 Table 4-A1) were analysed. Phylogenetic analyses using the maximum likelihood (ML) method were carried out with MEGA version 6.0 (Tamura et al. 2013). The nucleotide substitution model that best fitted the data set was identified using Model-Test in MEGA6. Based on the Akaike Information Criterion, the Jukes–Cantor model was identified as the best model. Gaps in the alignment were treated as missing characters. Bootstrap iteration was based on 1000 replicates and the percentage value was indicated at each node. *Neospora caninum* (GenBank accession number: AF038860.1) and *Toxoplasma gondii* (EU025025.1) were used as out-group species to root the tree.

### Statistical analysis

Data generated were analysed using the Statistical Package for the Social Sciences (SPSS) software version 25.0. Descriptive statistics were used to determine the prevalence of detected *Eimeria* species.

### Ethical consideration

The protocol for this study was approved by the University of KwaZulu-Natal Animal Research Ethics Committee and assigned the reference number AREC/058/017D.

## Results

### Polymerase chain reaction amplification and microscopic unsporulated oocyst detection

Among broiler and free-ranging chickens, 19 out of 41 pens (46.3%) and 25 out of 42 samples (59.5%) were positive for *Eimeria* infection (Figure 1). The highest level of *Eimeria* infection was observed in the following locations in both broiler and free-ranging chickens as shown in Figure 2: Phoenix (7/41; 17.1%), Scottburg (4/41; 9.8%), Shongweni (8/42; 19%), Port Sherpstone (7/42; 16.7%) and Maphumulo (7/42; 16.7%).

**FIGURE 1:**
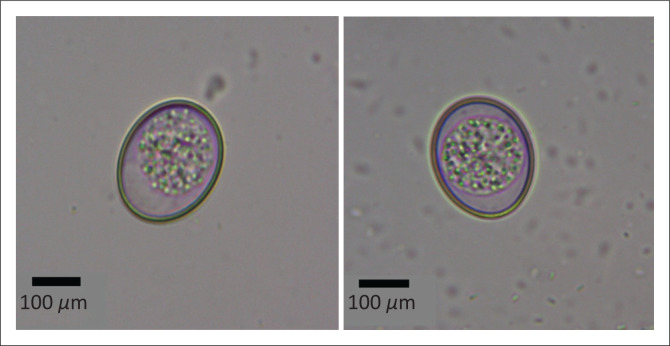
Images of unsporulated *Eimeria* oocysts detected in faecal samples from infected farms.

**FIGURE 2:**
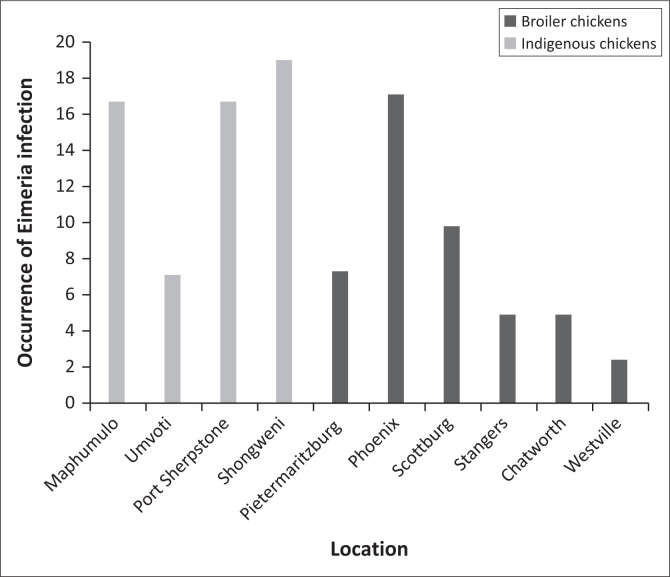
Occurrence of *Eimeria* infection in different locations in KwaZulu-Natal.

**FIGURE 3:**
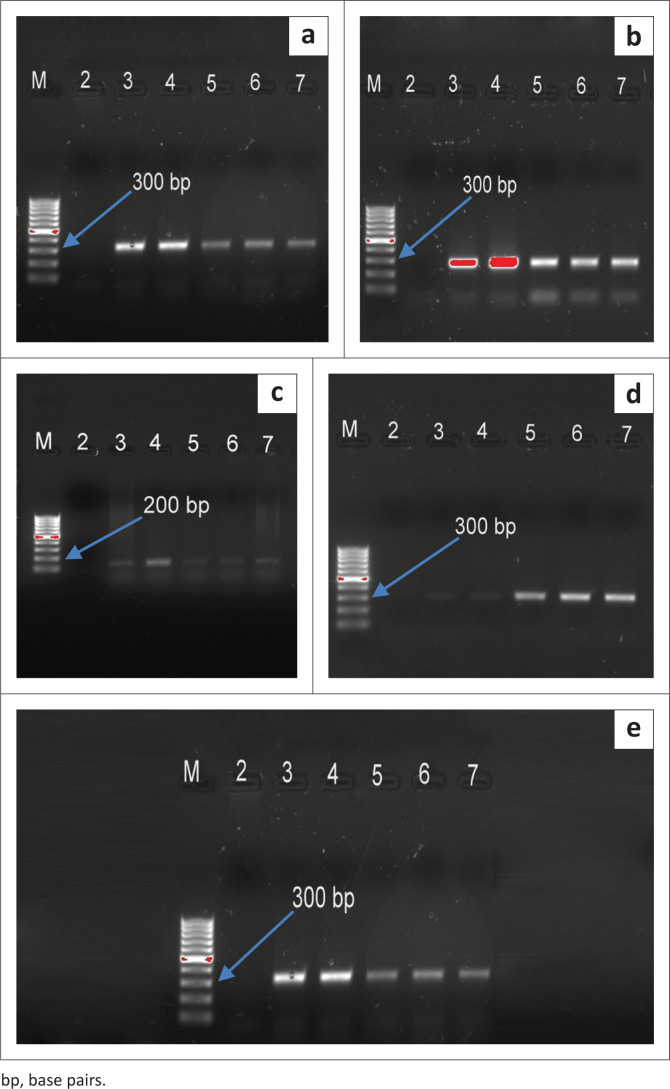
Amplification of *Eimeria* species by polymerase chain reaction. M: 100 bp DNA marker; L2: negative control; L3–7: samples. (a) *Eimeria mitis* 328 bp; (b) *Eimeria tenella* 278 bp; (c) *Eimeria maxima* 145 bp; (d) *Eimeria acervulina* 321 bp; (e) *Eimeria brunetti* 311 bp.

**FIGURE 4:**
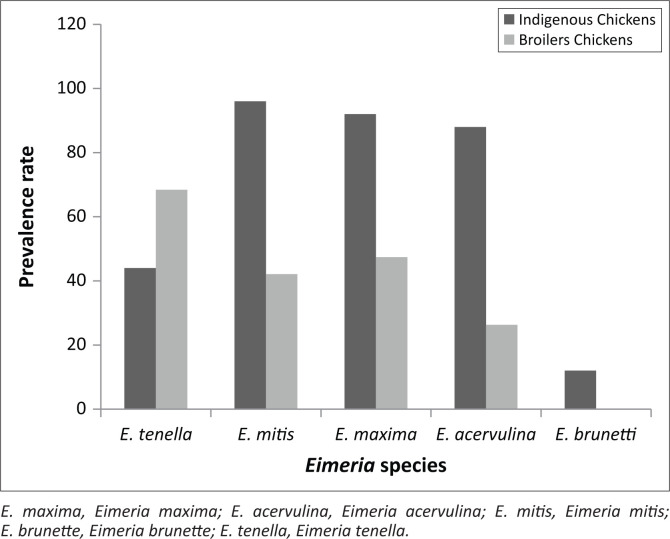
Prevalence of *Eimeria* species in both broilers and free-range chickens in KwaZulu-Natal.

Using the species-specific nested PCR assay, five *Eimeria* species were identified (*E. tenella, E. maxima, E. acervulina, E. brunetti* and *E. mitis*) in all screened samples (Figure 3). In broiler farms, *E. tenella* had the highest prevalence (13/19; 68.4%), followed by *E. maxima* (9/19; 47.4%) based on pens which were positive. However, in free-ranging chickens, *E. mitis* (24/25; 96%) and *E. maxima* (23/25; 92%) had the highest prevalence. The lowest prevalence was observed for *E. acervulina* (5/19; 26.3%) and *E. brunetti* (3/25; 12%) in broiler and free-ranging chickens, respectively (Figure 4).

### DNA amplification of Eimeria species

The most common mixed species combinations detected in broiler and free-ranging chicken faecal samples were *E. tenella* + *E. maxima* (4/19; 21.1%) and *E. mitis* + *E. maxima* + *E. acervulina* (11/25; 44%), respectively. Other combinations were *E. mitis* + *E. maxima* (2/19; 10.5%), *E. tenella* + *E. mitis* (1/25; 4%), *E. acervulina* + *E. maxima* (1/25; 4%), *E. acervulina* + *E. tenella* (2/19; 10.5%), *E. mitis* + *E. tenella* (3/19; 15.8%), *E. acervulina* + *E. tenella* + *E. maxima* (1/19; 5.3%), *E. acervulina* + *E. mitis* + *E. tenella* (2/19; 10.5%), *E. tenella* + *E. acervulina* + *E. mitis* + *E. maxima* (7/25; 28%), *E. tenella* + *E. mitis* + *E. brunetti* + *E. maxima* (1/25; 4%), *E. acervulina* + *E. mitis* + *E. tenella* + *E. maxima* (3/19; 15.8%) and *E. tenella* + *E. acervulina* + *E. mitis* + *E. maxima* + *E. brunetti* (2/25; 8%). Overall, among the broiler farms, Scottburg farm had the highest prevalence level of mixed species (*E. acervulina* + *E. mitis* + *E. tenella* + *E. maxima*; 75%), whilst mixed species (*E. acervulina* + *E. mitis* + *E. maxima*) with a prevalence of 44% was the highest among all locations with the free-range chickens. Cryptic *Eimeria* OTUs were not detected in all the samples screened.

### Internal transcribed spacer-1 sequence analysis

Internal transcribed spacer-1 sequences of *E. mitis, E. maxima, E. tenella, E. acervulina* and *E. brunetti* from this study showed high homology with sequences from *Eimeria* species present in the GenBank as follow: 90% – 93% identity for *E. mitis*, 99.31% for *E. maxima*, 99% – 100% for *E. tenella*, 99.38% for *E. acervulina* and 100% for *E. brunetti.* The overall mean genetic distance within *Eimeria* species isolates from KwaZulu-Natal in South Africa calculated by ML (Tamura 3-parameter model) with 1000 bootstrap replicates was 1.14 ± 0.08. Mean genetic distance per species was as follows: *E. mitis* (0.13 ± 0.014), *E. maxima* (0.09 ± 0.020), *E. tenella* (0.09 ± 0.012), *E. acervulina* (0.02 ± 0.005) and *E. brunetti* (0.02 ± 0.006).

### Phylogenetic analysis of internal transcribed spacer-1 sequences

**FIGURE 5:**
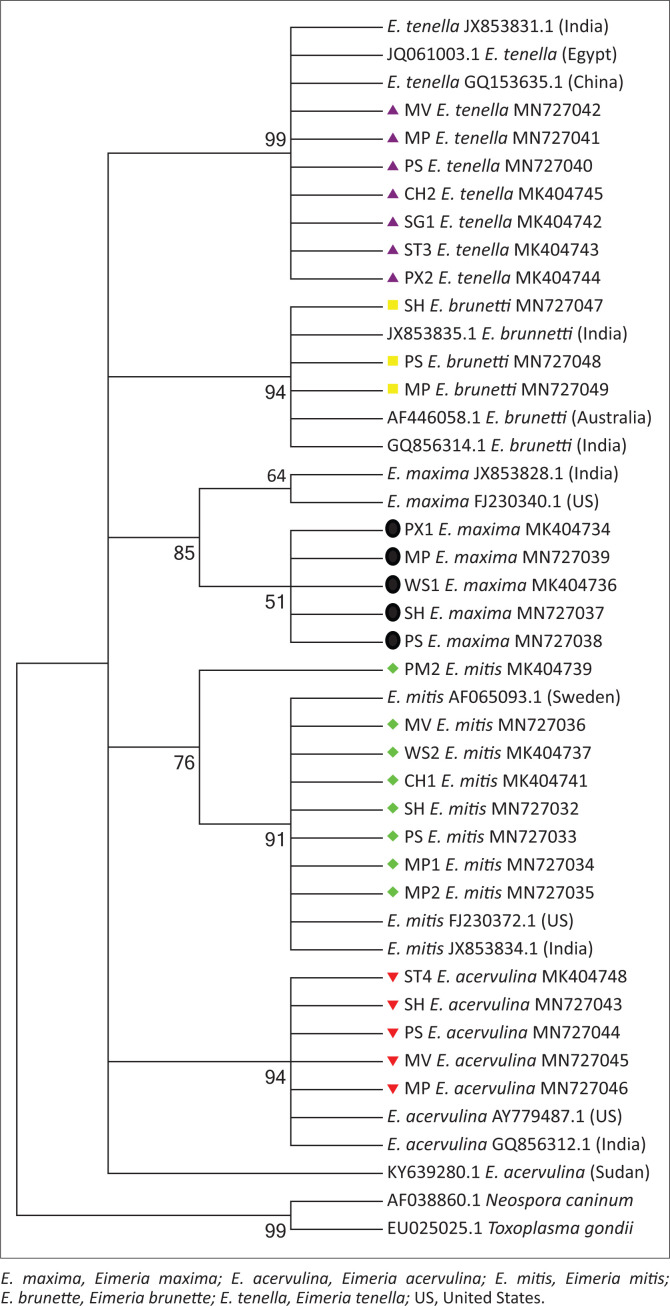
Maximum likelihood tree (Tamura-3 model) of internal transcribed spacer-1 sequences of *Eimeria* species. Percentage of bootstrap (1000 replicate) values is indicated in each node. The scale bar indicates sequence substitution per site. Sequences in this study are in different colours and shapes.

Maximum likelihood with the Jukes–Cantor model was used to create the phylogenetic tree (Figure 5) of the 28 ITS-1 sequences generated in this study, together with reference *Eimeria* ITS-1 sequences of American, Chinese, Indian, Australian and Swedish isolates. Irrespective of their geographical locations, the ITS-1 sequences of all five species clustered in distinct clades. Among the *E. tenella* clade, all the seven *E. tenella* sequences from this study clustered with *E. tenella* sequences from China, Egypt and India with a very strong support. Similarly, all the five and eight sequences of *E. acervulina* and *E. mitis* from this study, respectively, clustered with *E. acervulina* and *E. mitis* sequences of America, India, Sudan and Sweden with a very strong support. All the *E. maxima* sequences from this study clustered with *E. maxima* sequences from America and India with low support. Within the *E. brunetti* clade, all the three sequences from this study clustered with *E. brunetti* sequences from India and Australia with a very strong support. Genetic distances between ITS-1 sequences of *Eimeria* isolates in this study and those of a public database were as follow: *E. mitis* (0.12 ± 0.013), *E. acervulina* (0.02 ± 0.005), *E. maxima* (0.07 ± 0.016), *E. tenella* (0.07 ± 0.010) and *E. brunetti* (0.01 ± 0.004).

## Discussion

Coccidiosis is an enteric disease that poses a threat to efficient poultry production (Ogedengbe, Hanner & Barta 2011), compromising economic productivity and chicken welfare. For effective diagnosis, control and epidemiology of the disease, the identification of specific species of *Eimeria* is essential. Understanding the occurrence of genetic diversity and regional population structure are important (Hamza, Al-Massodi & Jeddoa 2015; Morris & Gasser 2006).

In this study, *Eimeria* infection had an overall prevalence of 46.3% (19 out of 41 pens) and 59.5% (25 out of 42 samples) across different farms and locations, which was higher than the 29.4% found among *Eimeria* parasites from KwaZulu-Natal and Limpopo (Malatji et al. 2016). However, it was lower than previous reports from other regions including Ethiopia (56%; Luu et al. 2013), Romania (91%; Gyorke et al. 2013), Anhui Province, China (87.75%; Huang et al. 2017) and two north Indian states (81.3%; Kumar et al. 2015b).

Molecular diagnosis using nested species-specific ITS-1 primers was used to identify five species of *Eimeria* (*E. tenella, E.maxima, E. acervulina, E. brunetti* and *E. mitis*) circulating in both commercial broiler and free-range chickens in KwaZulu-Natal province. This is similar to the study of Debbou-Iouknane, Benbarek and Ayad (2018), who reported the same five species of *Eimeria* among broilers farms in Bejaia region of Algeria. The prevalence of one or more species of *Eimeria* in broiler farms in this study could be influenced by the different anticoccidial used in various farms (Carvalho et al. 2011), although our study did not document anticoccidial use in the farms.

The most prevalent species among broiler farms in this study was *E. tenella* (68.4%), which is in agreement with other studies that have reported a high prevalence that ranges from 80.67% to 100% in Anhui Province, China, Trinadad and Indonesia (Brown et al. 2018; Hamid et al. 2018; Huang et al. 2017). The high prevalence of *E. tenella* poses a major concern to the health status of chickens because it is associated with caecal lesions causing haemorrhage, oedema and anaemia (Iacob & Duma 2009). However, *E. mitis* (96%) had the highest prevalence among free-ranging village chickens in this study. The reason for this is unclear as it is contrary to reports of most studies where *E. acervulina* and *E. tenella* are known to be highly prevalent in most farms because of their high reproductive potentials (Williams 2001).

Co-infection with multiple *Eimeria* species is a common finding in many poultry farms (Aarthi et al. 2010; Haug et al. 2008). We also found multiple infections (57.9% and 100%) to be common in both chicken types, with two or more species among the samples examined. *Eimeria tenella* + *E. maxima* (21.1%) and *E. mitis* + *E. maxima* + *E. acervulina* (44%) were the most common co-infections. This is in line with different studies which reported the frequency of *E. maxima* in most mixed species infection (Kaboudi, Umar & Munir, 2016).

Mixed infections among *Eimeria* species poses a challenge to the control of coccidiosis in chickens as it can increase pathogenicity of the disease among birds (Jekins et al. 2008). It could also serve as a potential threat to the effectiveness of anticoccidial vaccine, and this has warranted the combination of different *Eimeria* strains in some species, such as *E. maxima*, in the design of anticoccidial vaccines.

The efficacy of anticoccidial vaccines is under threat, especially with the recent upsurge of new *Eimeria* variants (OTUs), which was first detected circulating among commercial birds in Australia (Cantacessi et al. 2008). The presence of these OTUs (OTUx, OTUy and OTUz) has also been reported across much of the Southern Hemisphere (Clark et al. 2016; Fornace et al. 2013; Jatau et al. 2016). In this study, none of the samples was positive for any of the three OTUs. This could be because of the geographical location of our study sample, which is on latitude 28°S. Although a study has reported the distribution of these cryptic species (OTUs) in the northern hemisphere (Jatau et al. 2016), a more elaborate study by Clark et al. (2016) in 20 different countries from five continents has opined that these OTUs are distributed towards the south of the 30°N latitude. The study reported eight different countries to be populated with OTUs with the following distribution: OTUz was found in all the eight countries south of the 30°N latitude and OTUx was detected south of 30°N in six out of the eight countries, whilst OTUx, OTUy and OTUz were only detected in Nigeria among all the African countries at the same geographical location (Clark et al. 2016).

Similarly, ITS-1 sequences belonging to five different *Eimeria* species were generated in this study. The similarity of the sequences generated in this study when compared with published *Eimeria* species sequences ranged from 90% to 93% in *E. mitis*, 99.31% in *E. maxima*, 99% to 100% in *E. tenella,* 100% in *E. brunetti* and 99.38% in *E. acervulina*. Although the ML tree, as shown in Figure 5, grouped all five species of *Eimeria* into five distinct clades, some level of variation existed within species of *Eimeria* in this study and that of the public database, as indicated by their mean genetic distances. The lowest genetic distance of 0.01 was observed among *E. brunetti* isolates. Similar ITS-1 sequence variations among *E. mitis, E. tenella* and *E. maxima* have also been reported by different authors (Bhaskaran et al. 2010; Kumar et al. 2015a; Lew et al. 2003; Thenmozhi, Veerakumari & Raman 2014).

In conclusion, this study characterised *Eimeria* species in broiler and free-range chickens based on molecular diagnostic techniques and determined their diversity in KwaZulu-Natal province. The study reports the presence of five *Eimeria* species (*E. tenella, E. maxima, E. acervulina, E. brunetti* and *E. mitis*), all of which are regarded as pathogenic. Although none of the chickens showed clinical signs of coccidiosis during sampling, the high prevalence of these pathogenic parasites in the study area suggests that subclinical infection is common in all infected chickens. Thus, effective control strategies remain imperative to curtail coccidial infection in poultry farms in the study areas. A survey on the types of anticoccidial used among commercial farms and their efficacy should be conducted to understand the impact of this disease. This will also help in the implementation of policies for the control of this disease in KwaZulu-Natal province.

## Figures and Tables

**FIGURE 1–A1:**
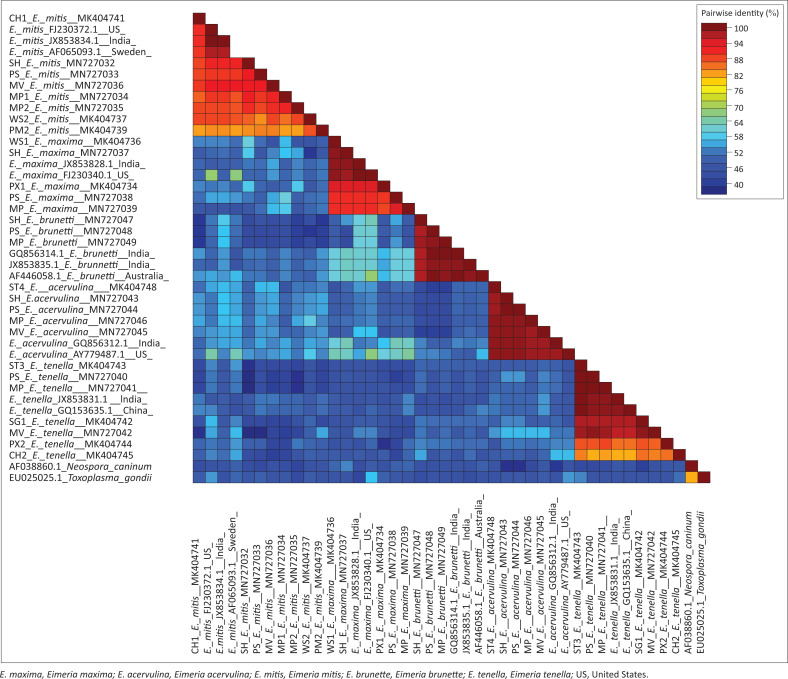
Pairwise percentage identity of ITS-1 sequences of different *Eimeria* species.

## Appendix 1

**TABLE 1–A1:** Summary of samples collected in broiler farms and the outcome of *Eimeria* detection.

Location	Farms	No of pen per farm	No of sample per farm	Age (Weeks)	Positive pen
Pietermaritzburg	A	5	40	3	0
	B	3	41	4	3
Phoenix	C	4	20	3	4
	D	3	15	4	2
	E	3	15	4	1
Scottburgh	F	4	25	4	4
Stanger	G	5	48	9	0
	H	4	22	10	1
	I	3	15	9	1
Chatsworth	J	3	28	10	1
	K	1	7	9	1
Westville	L	3	24	12	1
**Total**	**12**	**41**	**300**		**19**

**TABLE 2–A1:** Summary of samples collected in free-range chickens and the outcome of *Eimeria* infection.

Location	No of chicken	No of sample per location	No of positive samples
Maphumulo	10	10	7
Umvoti	10	10	3
Port Sherpstone	10	9	7
Shongweni	10	13	8
**Total**	**40**	**42**	**25**

**TABLE 3–A1:** Summary of samples collected in free-range chickens and the outcome of *Eimeria* infection.

Serial No	Sequence ID	Species	GenBank accession no.
1	PX1	*Eimeria maxima*	MK404734
2	WS1	*Eimeria maxima*	MK404736
3	WS2	*Eimeria mitis*	MK404737
4	PM2	*Eimeria mitis*	MK404739
5	CH1	*Eimeria mitis*	MK404741
6	SG1	*Eimeria tenella*	MK404742
7	ST3	*Eimeria tenella*	MK404743
8	PX2	*Eimeria tenella*	MK404744
9	CH2	*Eimeria tenella*	MK404745
10	ST4	*Eimeria acervulina*	MK404748
11	SH	*Eimeria mitis*	MN727032
12	PS	*Eimeria mitis*	MN727033
13	MP1	*Eimeria mitis*	MN727034
14	MP2	*Eimeria mitis*	MN727035
15	MV	*Eimeria mitis*	MN727036
16	SH	*Eimeria maxima*	MN727037
17	PS	*Eimeria maxima*	MN727038
18	MP	*Eimeria maxima*	MN727039
19	PS	*Eimeria tenella*	MN727040
20	MP	*Eimeria tenella*	MN727041
21	MV	*Eimeria tenella*	MN727042
22	SH	*Eimeria acervulina*	MN727043
23	PS	*Eimeria acervulina*	MN727044
24	MV	*Eimeria acervulina*	MN727045
25	MP	*Eimeria acervulina*	MN727046
26	SH	*Eimeria brunetti*	MN727047
27	PS	*Eimeria brunetti*	MN727048
28	MP	*Eimeria brunetti*	MN727049

PX, Phoenix; WS, Westville; PMB, Pietermaritzburg; CH, Chatsworth; SG, Stanger; ST, Scottburg; PS, Port Sherpstone; MV, Umvoti; MP, Maphumulo; SH, Shongweni.

**TABLE 4–A1:** ITS-1 sequences of *Eimeria* species downloaded from GenBank.

No	Species	GenBank accession number	Origin of isolates
1	*E. mitis*	FJ230372.1	America
2	*E. mitis*	JX853834.1	India
3	*E. mitis*	AF065093.1	Sweden
4	*E. maxima*	JX853828.1	India
5	*E. maxima*	FJ230340.1	America
6	*E. tenella*	GQ153635.1	China
7	*E. tenella*	JX853831.1	India
8	*E. tenella*	JQ061003.1	Egypt
9	*E. acervulina*	AY779487.1	America
10	*E. acervulina*	GQ856312.1	India
11	*E. acervulina*	KY639280.1	Sudan
12	*E. bruneeti*	AF446058.1	Australia
13	*E. brunetti*	GQ856314.1	India
14	*E. brunetti*	JX853835.1	India

*E. maxima, Eimeria maxima; E. acervulina, Eimeria acervulina; E. mitis, Eimeria mitis; E. brunette, Eimeria brunette; E. tenella, Eimeria tenella;* US, United States.
